# Supplementary material for: The evolution and role of the hyposphene-hypantrum articulation in Archosauria: phylogeny, size and/or mechanics?
Source: R Soc Open Sci. 2019 Oct 2;6(10):190258. doi: 10.1098/rsos.190258 (PMC6837189; doi:10.1098/rsos.190258)
Supplement: Linear Regression R code [file rsos190258supp4.docx]

**R code for logistic regression:**

library(rms) #call up package

library(pscl) #call up package (make sure installed first, from Tools ->Install packages)

cand<-read.csv("**FILE NAME**") #call up file

attach(cand) #attach names of column

fit <- glm(hypo~femlen,family=binomial(logit)) #logistic regression

plot(hypo~femlen, xlab="Femoral Length (mm)", ylab="", cex=1.3,pch=21, col="black", bg="blue") #plot

boxplot(fem0, add=T, boxwex=0.2, horizontal = T, at=0.1, col=rgb(1,0.498,0,0.4))

boxplot(fem1, add=T, boxwex=0.2, horizontal = T, at=0.9, col=rgb(0,0,1,0.4))

curve(predict(fit,data.frame(femlen=x),type="resp"),add=TRUE, lwd=2) #plot logistic line

points(fem0,zero,cex=1.3,pch=21, col="black", bg="darkorange1") #plot zeroes as orange

points(fem1,one,cex=1.3,pch=21, col="black", bg="blue") #plot ones as blue points on top of line

#legend(425,.6, c("Hyposphene-hypantrum present", "Hyposphene-hypantrum absent"), bty="n", pch=21, pt.bg=c("blue","darkorange1"), pt.cex = 1.5) # add legend

fit2<-lrm(formula = hypo~femlen) #logistic regression (but will give you stats)

fit2 #see stats--Nagelkerke R-squared index

pR2(fit) #more stats--McFaddens' R-squared

**R output for Avemetarsalia:**

Logistic Regression Model

lrm(formula = hypo ~ femlen)

Model Likelihood Discrimination Rank Discrim.

Ratio Test Indexes Indexes

Obs 50 LR chi2 40.11 R2 0.772 C 0.974

0 16 d.f. 1 g 10.842 Dxy 0.949

1 34 Pr(> chi2) <0.0001 gr 51144.394 gamma 0.949

max |deriv| 0.003 gp 0.405 tau-a 0.421

Brier 0.074

Coef S.E. Wald Z Pr(>|Z|)

Intercept -4.2991 1.5389 -2.79 0.0052

femlen 0.0181 0.0073 2.48 0.0132

>

> pR2(fit) #more stats--McFaddens' R-squared

llh llhNull G2 McFadden r2ML r2CU

-11.2867896 -31.3434729 40.1133665 0.6398998 0.5516887 0.7720629

**R output for Pseudosuchia:**

Logistic Regression Model

lrm(formula = hypo ~ femlen)

Model Likelihood Discrimination Rank Discrim.

Ratio Test Indexes Indexes

Obs 20 LR chi2 19.09 R2 0.831 C 0.979

0 8 d.f. 1 g 7.128 Dxy 0.958

1 12 Pr(> chi2) <0.0001 gr 1246.269 gamma 0.958

max |deriv| 9e-05 gp 0.479 tau-a 0.484

Brier 0.067

Coef S.E. Wald Z Pr(>|Z|)

Intercept -9.2579 5.3061 -1.74 0.0810

femlen 0.0338 0.0187 1.81 0.0704

>

> pR2(fit) #more stats--McFaddens' R-squared

llh llhNull G2 McFadden r2ML r2CU

-3.9143823 -13.4602333 19.0917021 0.7091891 0.6150282 0.8314261
